# Overexpression of Cu-Zn SOD in *Brucella abortus* suppresses bacterial intracellular replication via down-regulation of Sar1 activity

**DOI:** 10.18632/oncotarget.24073

**Published:** 2018-01-10

**Authors:** Xiaofeng Liu, Mi Zhou, Yanling Yang, Jing Wu, Qisheng Peng

**Affiliations:** ^1^Key Laboratory of Zoonosis, Ministry of Education, Institute of Zoonosis, Jilin University, Changchun 130062, China; ^2^Tumor Hospital of Jilin Province, Changchun 130021, China; ^3^Changchun Medical College, Changchun 130031, China; ^4^Institute of Special Wild Animal and Plant Science, Chinese Academy of Agricultural Sciences, Changchun 130122, China; ^5^School of Nursing, Gansu University of Chinese Medicine, Lanzhou 730000, China

**Keywords:** Brucella, SOD, Sar1

## Abstract

*Brucella* Cu-Zn superoxide dismutase (Cu-Zn SOD) is a periplasmic protein, and immunization of mice with recombinant Cu-Zn SOD protein confers protection against *Brucella abortus* infection. However, the role of Cu-Zn SOD during the process of *Brucella* infection remains unknown. Here, we report that Cu-Zn SOD is secreted into culture medium and is translocated into host cells independent of type IV secretion systems (T4SS). Furthermore, co-immunoprecipitation and immunofluorescence studies reveal that *Brucella abortus* Cu-Zn SOD interacts with the small GTPase Sar1. Overexpression of Cu-Zn SOD in *Brucella abortus* inhibits bacterial intracellular growth by abolishing Sar1 activity in a manner independent of reactive oxygen species (ROS) production.

## INTRODUCTION

The genus *Brucella* is a gram-negative facultative intracellular pathogen that causes brucellosis in humans and many animals, including cows, goats, sheep, dogs, pigs and et al. Brucellosis is one of the zoonotic diseases, which causes abortion and sterility in animals, and debilitating disorders in humans. Difficulties in the production of a safe and effective vaccine are the cause of the high levels of infectivity of *Brucella*. Better understanding of the *Brucella* proteins that support *Brucella* intracellular growth is critical to design a safe and effective brucellosis vaccine [1].

The *Brucella* Cu-Zn superoxide dismuatse (Cu-Zn SOD) is an 18.5 KD periplasmic protein encoded by *Brucella* sodC gene, and an appropriate immune response to this protein confers protection against *B. abortus* challenge in a mouse model [2]. Intramuscular administration of a plasmid vector coding for Cu-Zn SOD confers protection in mice and strong immune responses in calves [3]. More importantly, overexpression of Cu-Zn SOD in a RB51 vaccine strain (RB51SOD strain) significantly increases its vaccine efficacy against *B. abortus* challenge and decreases its survival rate in macrophages [4]. However, the mechanism by which overexpressed Cu-Zn SOD mediates bacterial intracellular replication remains unknown. As the virulence of *Brucella* depends on survival and replication properties in host cells [1], it will be important to determine the role of Cu-Zn SOD in regulating *Brucella* intracellular growth for designing a safe vaccine in the future.

The virB operon-encoded Type IV Secretion System (T4SS) is required for multiplication and intracellular survival, and the effectors, which are secreted by T4SS, play a critical role in intracellular trafficking of *Brucella spp* [5]. Cu-Zn SOD protein is able to be secreted in bacteriological culture supernatant [6]. However, we do not know whether Cu-Zn SOD is translocated into host cells. Because *Brucella* effectors, such as RicA and SepA [7, 8], are periplasmic proteins and also can be secreted into culture supernatant, it is necessary to investigate whether Cu-Zn SOD is a VirB-dependent or independent effector, which will benefit for further understanding the role of Cu-Zn SOD in mediating intracellular bacterial growth.

In this study, we report that Cu-Zn SOD protein is a VirB-independent effector. Overexpressed Cu-Zn SOD protein in *B. abortus* strain 2308 inhibits bacterial intracellular grow via down-regulation of host Sar1 activity.

## RESULTS

### The release of Cu-Zn SOD into the culture supernatant of *B. abortus* is a VirB-independent process

**Figure 1:**
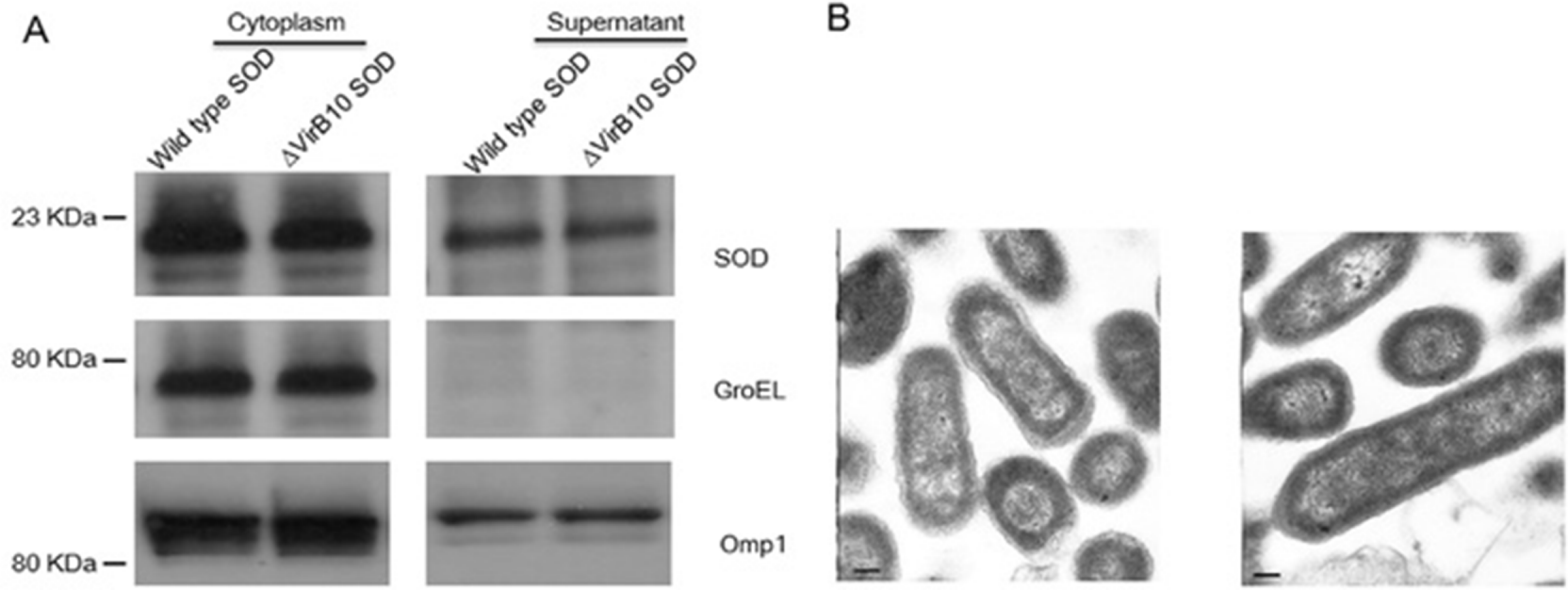
The secretion of Cu-Zn SOD is a VirB-independent process (**A**) Bacterial supernatants of 10 ml cultures of *B. abortus* strain 2308 and *B. abortus virB10* carrying the plasmid coding for Cu-Zn SOD -3Flag(ΔVirB10 SOD) were precipitated by TCA. Anti-FLAG, anti-GroEL and anti-OMP1 monoclonal antibodies were used to immunoblot bacterial cytoplasmic fractions (left part) and concentrated culture supernatants (right part). (**B**) Electron microscopy of outer membrane of wild type strain (left part) or *B. abortus* overexpressed Cu-Zn SOD-3Flag (wild type SOD) (right part). The scale bar is 100 nm.

Secretion of Cu-Zn SOD by *B. abortus* has been documented in previous studies [6]. However, the role of the VirB T4SS in mediating Cu-Zn SOD secretion remains unknown. To determine if Cu-Zn SOD secretion is dependent on the VirB T4SS, the plasmids expressed Cu-Zn SOD-Flag fused protein with Flag tag at C-terminus were generated. The plasmids were transfected into the *B. abortus* 2308 wild type strain or a *B. abortus* 2308 ∆virB10 strain, respectively. After growth in the tryptic soy broth, the Cu-Zn SOD proteins in the bacterial pellets, as well as in the culture supernatants precipitated with TCA, were analyzed by Western-blotting with the anti-Flag antibody. Our data show that the Cu-Zn SOD proteins were detected in the concentrated supernatant of wild type strain and ∆virB10 strain. Cytoplasmic control protein, GroEL, was not detected in the supernatant; while periplasm protein Omp1, used as a secreted protein positive control, was detected in wild type strain and ∆virB10 strain (Figure 1A). This result suggests that VirBT4SS is not required for secretion of Cu-Zn SOD in the culture medium.

Zhu, *et al.* has reported that overexpressed Cu-Zn SOD protein by *Brucella* vaccine strain RB51 damages the integrity of the outer membrane of RB51 *Brucella* cells [4]. To determine if secretion of Cu-Zn SOD protein in *B. abortus* 2308 results from leakage of damaged outer membranes, we investigated membrane integrity with transmission electron microscope analysis of wild type *B. abortus* 2308 and 2308 overexpressing Cu-Zn SOD. Compared with the wild type, no physical damage was observed in the strain overexpressing Cu-Zn SOD (Figure 1B). Taken together, these data indicate that the release of Cu-Zn SOD into the culture medium is not a result of physical damage of cell membrane but through a T4SS-independent system.

### Cu-Zn SOD is a VirB T4SS-independent effector

**Figure 2:**
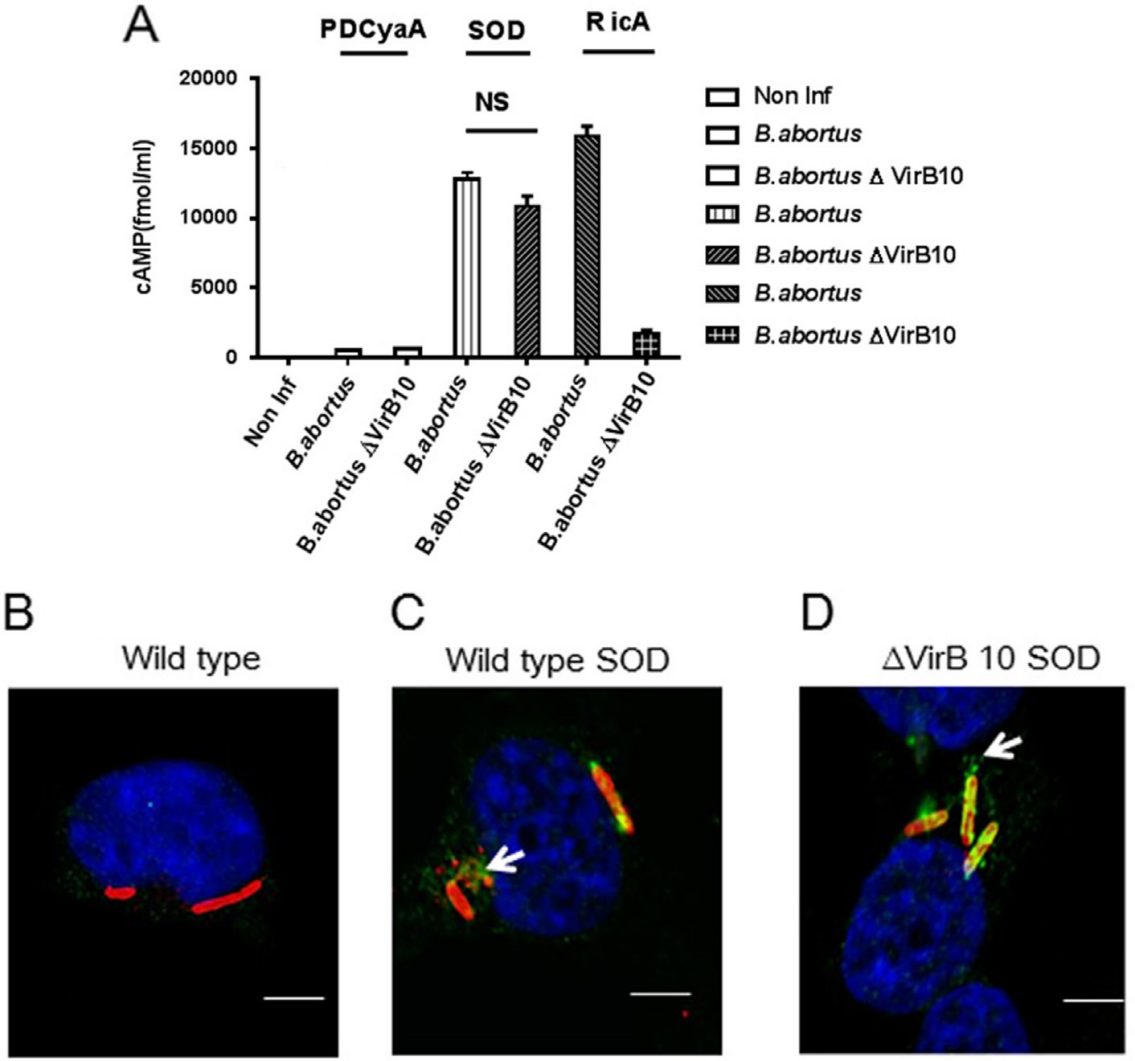
VirB-independent translocation of Cu-Zn SOD into the cytoplasm of host cells (**A**) Intracellular cAMP levels in J774.A1 cells infected with *B. abortus* or *B. abortus* ∆virB10 expressing Cu-Zn SOD-3Flag-CyaA fusion proteins were measured after a 6-hour infection. *B. abortus* and *B. abortus* ∆VirB10 containing the CyaA domain alone (pDCyaA) were designed as negative controls. *B. abortus* and *B. abortus* ∆VirB10 expressing RicA-3Flag-CyaA fusion proteins were designed as positive controls. Intracellular cAMP levels were also quantified in non-infected cells. Mean and SEM are shown for one representative out of three independent experiments. (**B**–**D**) Representative confocal micrographs of BMDM cells infected with wild type SOD strain(C) or ΔVirB10 SOD strain (D). BMDM cells infected with wild type *B. abortus* was designed as a negative control (B).At 6 h post-infection, cells were fixed and processed for immunostaining as described in experimental procedures. Red, *Brucella*; green, Flag; blue, cell nucleus.

To test whether Cu-Zn SOD is a VirB TSS4-dependent effector during infection, the *B. pertussis* calmodulin-dependent adenylate cyclase (CyaA) translational fusions with Cu-Zn SOD was generated in plasmids pBBR1MCS, and introduced into either wild type 2308 or the ∆virB10 mutant strain [9]. We used the RicA-CyaA fusion protein as a positive control, as it has been previously reported to be translocated into host cells [7]. The CyaA alone was used as a negative control. If Cu-Zn SOD–CyaA is translocated to J774.A1 cytoplasm, delivery of the corresponding CyaA fusion protein into the cytosol would lead to calmodulin-dependent activation of adenylate cyclase and enzymatic conversion of ATP to cAMP. The cytoplasmic cAMP concentrations were subsequently measured by quantitative ELISA [9, 10]. As shown in Figure 2A, cAMP activity was detected at 6 h post-infection in the presence of RicA-CyaA fusion protein in *B. abortus* compared with non-infected cells. CyaA control protein failed to induce cytosolic cAMP following infection with *B. abortus*. J774.A1 cells infected with wild-type *Brucella* carrying Cu-Zn SOD-CyaA had significantly increased cytosolic cAMP production, indicating that Cu-Zn SOD can be translocated into J774.A1 cells. Interestingly, Cu-Zn SOD–CyaA also significantly increased cAMP in J774.A1 cells infected with virB10 mutant *Brucella* strains. These data indicate that translocation of Cu-Zn SOD into host cells is VirB T4SS-independent.

To characterize Cu-Zn SOD translocation into host cells via virB-independent manner, bone marrow derived macrophages (BMDMs) infected with wild type and virB10 mutant expressing Cu-Zn SOD-Flag were examined by confocal microscopy with an anti-Flag antibody. As *Brucella* is unable to be stained with anti-Flag antibody [9], Flag staining is marker of protein translocation from the bacteria into host cells. Compared to BMDMs infected with wild-type *B. abortus* (Figure 2B), Cu-Zn SOD is detected in the cytosol of BMDMs infected with *B. abortus* overexpressing Cu-Zn SOD (Figure 2C). Consistent with the assay of cytoplasmic cAMP, Flag staining of host cells was also observed in virB10-deficient *Brucella* infection (Figure 2D). Taken the data together, these results demonstrate that Cu-Zn SOD can be translocated into host cells in a VirB T4SS-independent manner.

### Overexpressed Cu-Zn SOD in *B. abortus* inhibits bacterial intracellular growth

**Figure 3:**
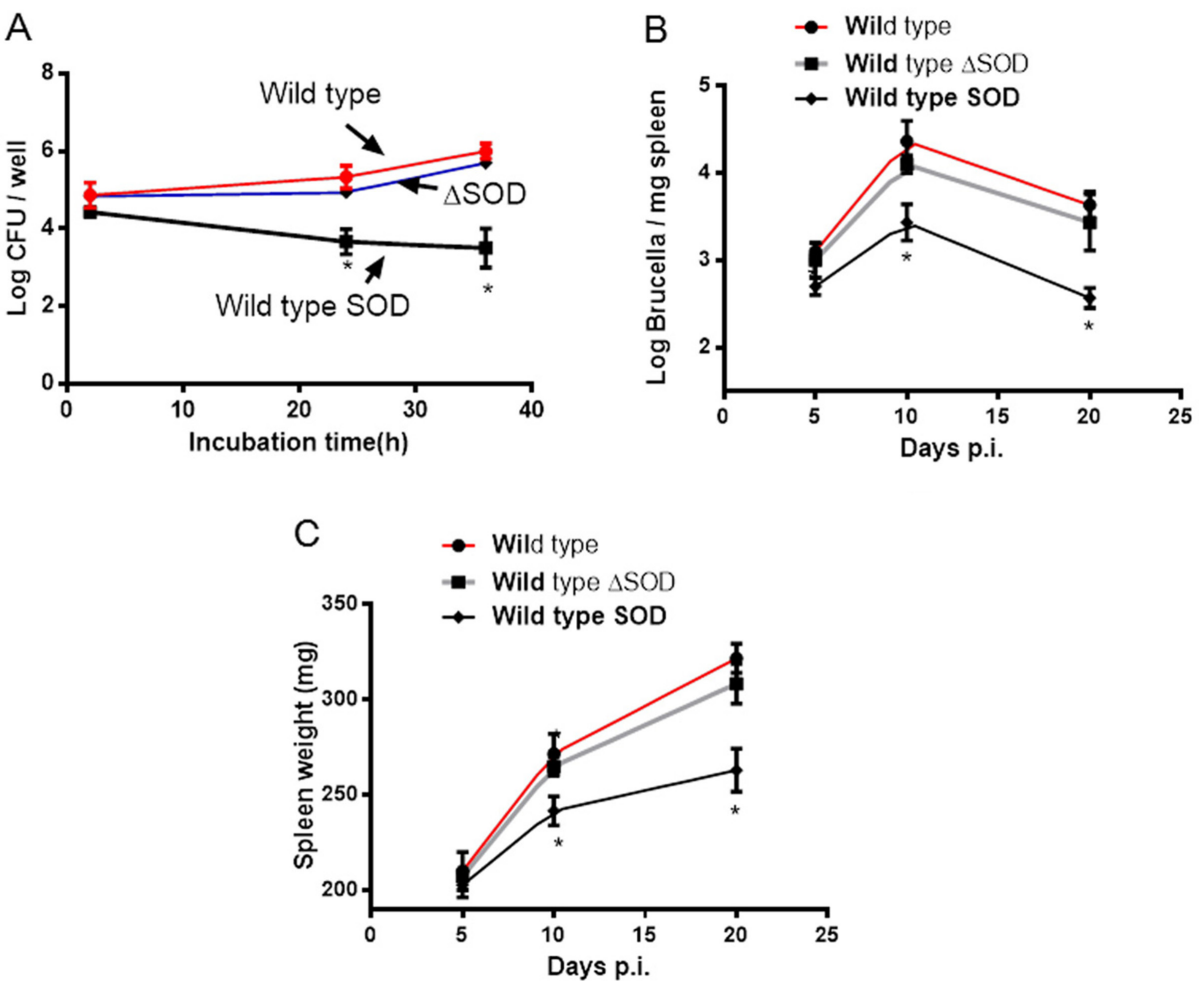
Overexpressed Cu-Zn SOD in *B. abortus* inhibits bacterial intracellular growth (**A**) BMDM cells were infected with *B. abortus* (wild type), Cu-Zn SOD mutant *B. abortus* (ΔSOD) or wild type SOD as indicated times. The number of viable bacteria was determined by counting CFU. (**B**) BALB/c mice were injected intraperitoneally with 10^5^ wild type, Δ SOD or wild type SOD strains. The number of viable bacteria was determined by counting CFU as the mean of LOG_10_^CFU^ per milligram of spleen. (**C**) Mean weights of BALB/c mice spleen inoculated with wild type, ΔSOD or wild type SOD strains. ^*^P < 0.05 versus control. Data are expressed as mean ± SEM.

The previous study demonstrated that Cu-Zn SOD deficiency in *B. abortus* did not affect bacterial growth within HeLa cells or J774.A1 macrophage cells [11]. Consistent with the report, we also did not observe the significant difference of intracellular growth in BMDMs in both wild type and Cu-Zn SOD mutant strains. However, the number of *B. abortus* overexpressing Cu-Zn SOD within BMDMs decreased significantly compared with wild type and Cu-Zn SOD mutant strains at 24 and 36 h post infection (Figure 3A).

Next, we tested whether the reduced intracellular survival of SOD-overexpressing *Brucella* in BMDMs translates to reduced virulence in mice. Wild type, Cu-Zn SOD mutant, and Cu-Zn SOD-overexpressing strains were used to infect mice and spleen colonization profiles were determined. As shown in Figure 3B, the numbers of wild type and SOD mutant strains recovered from the spleens of mice were statistically higher than SOD-overexpressing strain at 10 and 20 days post infection. Meanwhile, mice infected with Cu-Zn SOD-overexpressing strain had significantly lower spleen weights than those infected with wild type and Cu-Zn SOD mutant strains at 10 and 20 days post infection (Figure 3C). These data suggest that overexpression of Cu-Zn SOD in *Brucella* can suppress bacterial replication within host cells.

### ROS and NO production in macrophages is not influenced by overexpression of Cu-Zn SOD in *B. abortus*

**Figure 4:**
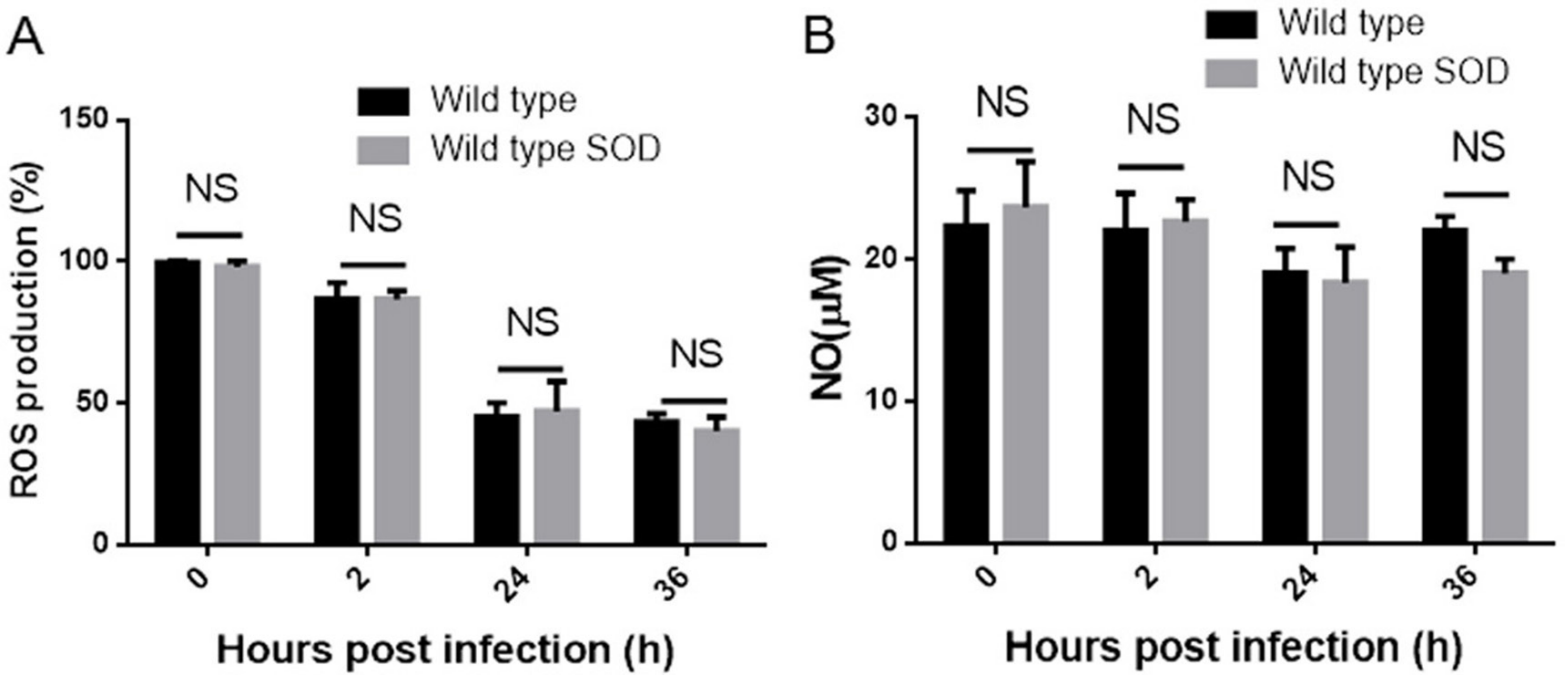
Overexpressed Cu-Zn SOD in *B. abortus* is unable to mediate the ROS and NO production in macrophages (**A**) BMDM cells were infected with wild type, or wild type SOD strains as the indicated times. The supernatant of infected cells were collected and filtered to determine NO production using the Greiss reagent. (**B**) A confluent monolayer of BMDM cells infected with wild type, or wild type SOD strains B. were measured at various time points. ROS production was assayed by DHE fluorescence.

SOD can catalyze the dismutation of superoxide (O^2−^) to hydrogen peroxide (H_2_O_2_), which can then be further detoxified to protect the intracellular bacteria from the respiratory burst of host macrophages [12]. Since we have shown that Cu-Zn SOD can be translocated into host cells, we hypothesized that overexpression of Cu-Zn SOD might suppress *Brucella* replication via regulating the production of reactive oxygen species (ROS). To determine the role of overexpression of Cu-Zn SOD in macrophages, the levels of ROS in BMDMs were measured at 2, 24 and 36 hours post infection. As indicated in Figure 4A, the production of ROS in BMDMs infected with Cu-Zn SOD-overexpressing *B. abortus* is similar to BMDMs infected with wild type strains. As nitric oxygen (NO) plays an important role in killing of the intracellular bacteria [13], we also investigate whether overexpressed Cu-Zn SOD participate in the production of NO. Consistent with the ROS assay, there was no significant change of NO production in BMDMs infected with wild type or Cu-Zn SOD-overexpressing strains (Figure 4B), suggesting that overexpression of Cu-Zn SOD does not mediate the NO production. Taken together, these data indicate that the production of ROS and NO are not involved in Cu-Zn SOD-mediated *Brucella* intracellular replication.

### SOD in *B. abortus* interacts with cytoplasmic Sar1 of host cells

**Figure 5:**
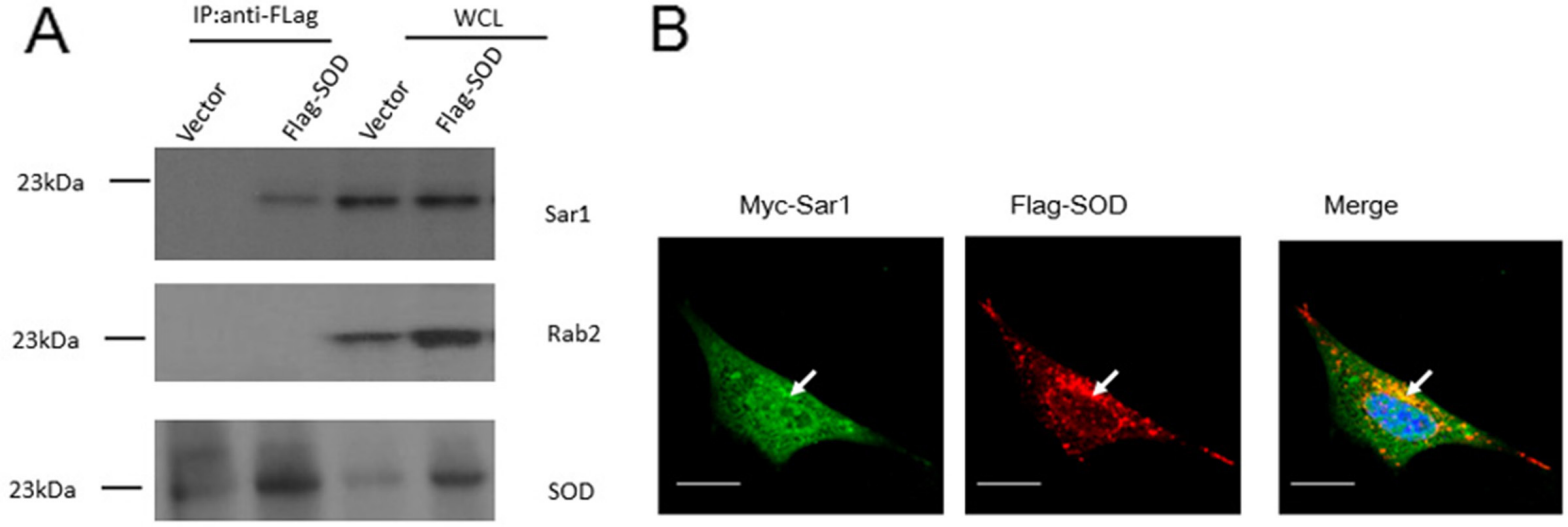
*B. abortus* Cu-Zn SOD interacts with Sar1 (**A**) Flag-tagged *B. abortus* Cu-Zn SOD or empty vector was expressed in HEK293 cells. Cu-Zn SOD was immunoprecipitated with anti-Flag antibody and co-precipitated Cu-Zn SOD, Sar1 and Rab2 were detected by immunoblotting with anti-Sar1 and anti-Rab2 antibodies (upper panel). The expression level of SOD, Sar1 and Rab2 were detected by immunoblotting the whole cell lysates with anti-Flag, anti-Sar1 and anti-Rab1 antibodies (down panel). (**B**) Cu-Zn SOD is co-localized with Sar1 in HeLa cells. Flag-Cu-Zn SOD was co-transfected with myc-Sar1 into HeLa cells for 36 h. In the left image, immunofluorescence was made with an anti-Myc antibody. In the middle image, immunofluorescence was made with the anti-Flag antibody. The right image was generated by merging the anti-Myc and anti-Flag signals. The scale bar is 20 um.

As Sar1 and small GTPase Rab2 are crucial for *Brucella* intracellular replication [14, 15], we hypothesized that Cu-Zn SOD overexpression might inhibit *Brucella* intracellular growth via interaction with Sar1 or Rab2. To test this hypothesis, we determined whether Cu-Zn SOD could bind to Sar1 or Rab2. 293T cells were transfected with plasmids encoding Flag-Cu-Zn SOD, and cell lysates were immunoprecipitated with anti-Flag antibody. Endogenous Sar1 and Rab2 co-precipitated with Cu-Zn SOD were detected by immunoblotting with anti-Sar1 and Rab2 antibodies. The data in Figure 5A showed that the SOD interacted with Sar1 but Rab2 did not. Constitutively active mutated Sar1(H79G) could still co-precipitate with Cu-Zn SOD (Supplementary Figure 2). To characterize the association of Sar1 with Cu-Zn SOD, we performed fluorescent microscopic studies of HeLa cells transfected with Myc-Sar1 and Flag- Cu-Zn SOD. Anti-Myc and anti-Flag staining of HeLa cells revealed a cytosolic co-localization as shown in the merged panel (Figure 5B). These data are consistent with Cu-Zn SOD associating with Sar1 in a cellular context.

### Overexpressed Cu-Zn SOD inhibits bacterial intracellular growth via abolishing the Sar1 activity

**Figure 6:**
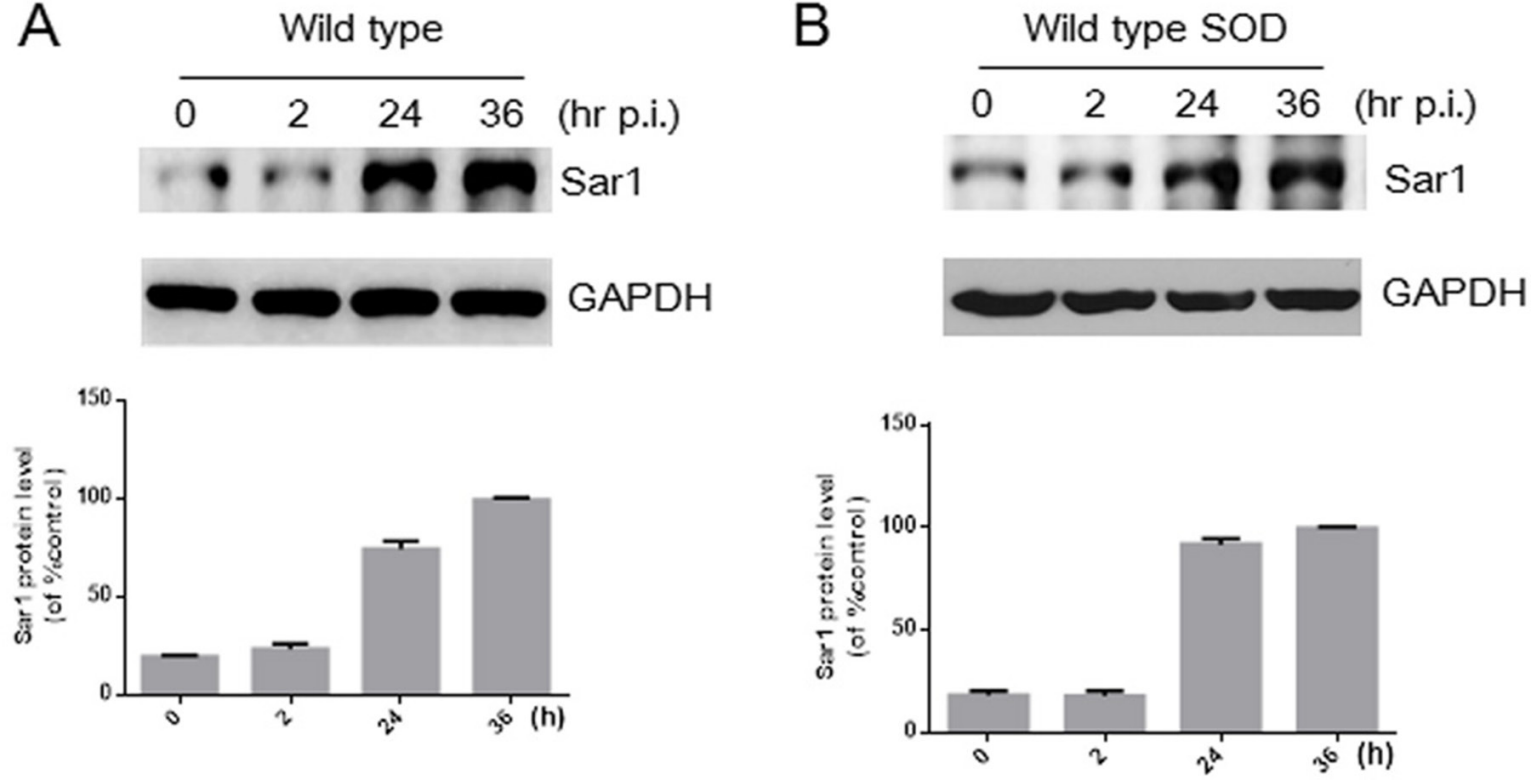
Infection with *B. abortus* or wild type SOD leads to the upregulation of Sar1 BMDM cells were infected with *B. abortus* (**A**) or wild type SOD (**B**) as indicated times. Cell lysates were collected and analyzed by Western blotting to determine the protein levels of Sar1. GAPDH was used for normalization. The blot is a representative of 3 independent experiments. The quantitative data for Sar1 expression are shown under Western blotting data. Data are expressed as mean ± SEM (*n* = 3).^*^P < 0.05 VS control.

*B. abortus* infection increases the expression level of Sar1 protein in HeLa cells [16]. To exclude the possibility that changes in Sar1 expression in HeLa cells contributes to different bacterial intracellular growth, protein levels of Sar1 were determined after infection wild type or Cu-Zn SOD-overexpressing strains. As shown in Figure 6A and 6B, Sar1 levels in HeLa cell infected with Cu-Zn SOD-overexpressing *Brucella* strains were also increased compared with the wild type strain, which is consistent with the results from Murata’s lab [16]. Additionally, our data showed that the expression level of another small G protein, Rab2, is not influenced during the stage of *Brucella* infection (Supplementary Figure 1). These data indicate infection-induced increase in Sar1 expression is not modulated by the overexpression of Cu-Zn SOD and likely do not participate in mediating bacterial replication.

**Figure 7:**
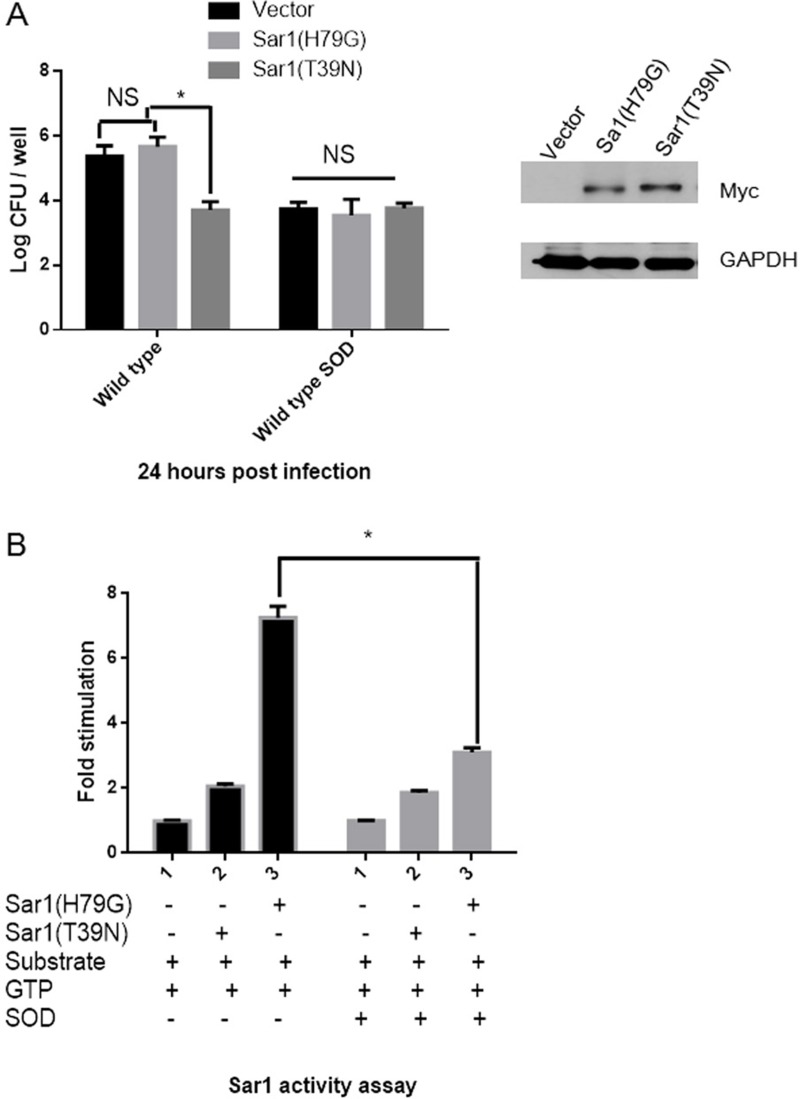
Overexpressed Cu-Zn SOD in *B. abortus* inhibits the Sar1 activity of host cells (**A**) HeLa cells were transfected with Myc-Sar1(H79G), Myc-Sar1(T39G) or vector for 24 h and then infected with *B. abortus* or wild type SOD for another 24 h. The number of viable bacteria was determined by counting CFU. ^*^P < 0.05 versus control. Data are expressed as mean ± SEM. (**B**) Membranes (75 μg) were incubated with GTP in the presence of recombinant His-Sar1(H79G) (8 μg), or recombinant His-Sar1(T39N) (8 μg), or recombinant His-Cu-Zn SOD, ethanol and radiolabeled liposomes for 60 min at 37°C. At the end of incubation, the lipids were extracted and the formation of phosphatidylethanol was determined using TLC. Results were averaged from two independent experiments and are presented as fold stimulation over control.

Next, we investigate whether Cu-Zn SOD suppresses *Brucella* intracellular growth by mediating the activity instead of the expression of Sar1. HeLa cells, in which *Brucella* can survive and replicate, were chosen as our cell model for this study. HeLa cells were transfected with empty vector, active Sar1(H79G) or inactive Sar1(T39N) [14]. Twenty-four hours after transfection, cells were infected with *Brucella*, and the number of intracellular *Brucella* was counted at 24 hours post infection. The number of intracellular *B. abortus* 2308 was significantly decreased in cells transfected with Sar1(T39N) compared with empty vector or Sar1(H79G) (Figure 7A). However, we did not observe the obvious difference of viable number of Cu-Zn SOD-overexpression *B. abortus* within HeLa cells transfected with empty vector, Sar1 (T39N) or Sar1 (H79G). These results imply that the decreased number of intracellular *Brucella* might be caused by Cu-Zn SOD expression, which interfere the activity of Sar1.

As Sar1 GTPase is a potent activator of phospholipase D (PLD) [17], we designed an *in vitro* PLD assay to determine whether Cu-Zn SOD is able to inhibit Sar1 activation of PLD. We incubated membranes in the presence of Sar1(H79G) or Sar1(T39N), Cu-Zn SOD, GTP, and a radiolabeled liposome substrate which is widely used to measure the activity of membrane-associated PLD [18]. In the absence of Cu-Zn SOD protein, active Sar1(H79G), but not inactive Sar1(T39N), stimulated the activity of PLD (Figure 7B). However, in the presence Cu-Zn SOD, PLD activity was significantly suppressed even in the presence of active Sar1(H79G). Furthermore, mutants of Cu-Zn SOD (K60R, D61A, and K63R), unable to bind to Sar1 (Supplementary Figure 3), failed to suppress intracellular bacterial growth (Supplementary Figure 4). Taken together; our data suggest that Cu-Zn SOD modulates bacterial replication in part by mediating the activity of Sar1.

## DISCUSSION

In this study, we show that the periplasmic protein Cu-Zn SOD in *B. abortus* is not only secreted into culture medium but also is able to be translocated into host cells in T4SS VirB-independent manner. Overexpressed-Cu-Zn SOD in *B. abortus* leads to suppression of *Brucella* survival. Mechanistically, we discovered a novel role for SOD in this process whereby overexpressed-Cu-Zn SOD can bind to the small GTPase protein Sar1, leading to inactivity of Sar1 and inhibition of bacterial intracellular growth.

One of the most important findings of this study is that Cu-Zn SOD is identified to act as T4SS VirB-independent effector in the process of *Brucella* infection. To explore the mechanism by which Cu-Zn SOD is translocated into host cells, we initially evaluated the effects of T4SS in mediating Cu-Zn SOD secretion in culture medium. Surprisingly, T4SS did not influence Cu-Zn SOD secretion from the periplasm into tryptic soy broth, indicating that T4SS is not involved in translocation of Cu-Zn SOD to host cells. Because some of *Brucella* effectors, such as BPE865, BPE159, BspD, BspG and BspK, have been reported to belong to T4SS VirB-independent effectors [9, 10], we propose that translocation of Cu-Zn SOD may not require a functional T4SS during the process of *B. abortus* infection. To test this hypothesis, a CyaA translocation reporter assay was utilized to detect *Brucella* SOD translocation at 6 h post infection. Compared with *Brucella* effector RicA, Cu-Zn SOD translocation was not significantly reduced in the ∆VirB10 mutant strain. This is line with confocal analysis of the secretion of the Cu-Zn SOD protein: VirB T4SS did not affect the translocation of Cu-Zn SOD to cytoplasm of host cells. These results suggested that the translocation of Cu-Zn SOD was independent of a functional T4SS. In future experiments, we will further investigate the presence of another unknown translocation system in *Brucella*.

Another important finding of this study is that overexpressed Cu-Zn SOD inhibits *B. abortus* intracellular growth via suppressing the activity of the small GTpase protein Sar1. Previous study showed that macrophages infected with *Brucella* RB51 Cu-Zn SOD strain exhibited a significantly lower number of surviving cells than RB51 strains [4]. However, the underlying mechanism remains elusive. To our knowledge, this study is the first to report that overexpressed Cu-Zn SOD plays an important role in mediating the activity of Sar1downstream of *Brucella* infection. Sar1 can be coopted by *Brucella* to promote bacterial replication inside host cells [14]. Our data showed that overexpressed Cu-Zn SOD did not influence the expression of Sar1 but did influence the activity of Sar1. The number of survival Cu-Zn SOD-overexpressed *Brucella* in HeLa cells, in which Sar1 active form (Sar1(H79G)) was expressed, is similar to HeLa cells, which was expressed Sar1 dead form (Sar1(T39G)). Moreover, our phospholipase D *in vitro* assay also confirm that Sar1 (H79G) incubation with Cu-Zn SOD can inhibit the activation of PLD in our membrane preparations. Considering that Cu-Zn SOD is able to interact with Sar1, these results suggest that the effects of Sar1 on *B. abortus* infection might be mediated by Cu-Zn SOD expression.

Previous study evaluating the function of *B. abortus* Cu-Zn SOD in experimentally infected mice or macrophages generated conflicting results to ours. Gee, et al. reported spleen or macrophage colonization profiles of *B. abortus* 2308 and a Cu-Zn SOD mutant *B. abortus* 2308 strain were significantly different, with the Cu-Zn SOD mutant strain displaying significant attenuation compared to wild type strain [12]. Careful comparison of the experimental procedures, suggests that this conflicting result may be due to different mice models used: BALB/c mice were used for infection in our study, whereas C57BL6J mice were used in the Gee’s lab. Indeed, some studies have demonstrated that C57BL6J mice are more sensitive for *Brucella* infection than BALB/c mice [19]. Therefore, it is not surprising that Cu-Zn SOD can work as antioxidant to protect *B. abortus* 2308 from the respiratory burst of macrophages of C57BL6J mice. However, our data shows that overexpression of Cu-Zn SOD in *B. abortus* 2308 is able to suppress the rate of bacterial intracellular replication, suggesting that Cu-Zn SOD has another function in mediating *Brucella* intracellular survival apart from working as antioxidant.

In conclusion, through identifying Cu-Zn SOD as a novel VirB T4SS-independent effector and characterizing the contribution to pathogenesis, this study expands our knowledge at the molecular level of how overexpressed Cu-Zn SOD in *Brucella* manipulates Sar1 activity of the host cell to inhibit its intracellular survival. Based on our data, it is tempting to speculate that overexpression of Cu-Zn SOD in *Brucella* vaccine strain could increase its vaccine effectiveness against virulent *B. abortus* challenge.

## MATERIALS AND METHODS

### Ethics statement

The animal protocol was reviewed and approved by the Jilin University Institute Animal Care and Use Committee. The present investigations conform to the Guide for the Care and Use of laboratory Animals published by the US National Institutes of Health (NIH Publication No.85-23, revised 1996).

### Antibodies

The primary antibodies used were: mouse monoclonal anti-Rab2 (Abcam), rabbit polyclonal anti-Sar1 (Abcam), mouse monoclonal anti-GAPDH (Cell Signaling Technology), mouse monoclonal anti-His(Sigma), mouse monoclonal anti-Flag (Sigma), mouse monoclonal anti-Myc (Cell Signaling Technology), mouse monoclonal anti-HA (Cell Signaling Technology), mouse monoclonal anti-GroEL(Santa Cruz) and mouse monoclonal anti-Omp1 (Santa Cruz). The secondary antibodies used for Western blotting were: Horse Radish Peroxidase (HRP)-conjugated Goat Anti-Mouse IgG (Santa Cruz) and HRP-conjugated Goat Anti-Rabbit IgG (Cell Signaling) antibodies. Normal rabbit IgG was purchased from Santa Cruz. The secondary antibodies used for immunofluorescence were: goat anti-rabbit IgG (H + L) secondary antibody, Alexa Fluor^®^ 488 conjugate (Invitrogen), and donkey anti-rabbit IgG secondary antibody and Alexa Fluor^®^ 594 conjugate (Invitrogen).

### *Brucella* and cell line

*B. abortus* strain 2308 was originally obtained from Ding’s laboratory at China Institute of Veterinary Drug Control (Beijing, China) and was grown either in tryptic soy broth (TSB) or in tryptic soy agar plates. *B. abortus* ∆VirB10 and ΔCu-Zn SOD strains were kindly provided by Zeliang Chen (Institute of Disease Control and Prevention, Academy of Military Medical Science, China). Cu-Zn SOD-expressing in *Brucella* was made by electroporating the wild type or ΔVirB10 strains with a pBBR1MCS-2 containing the Cu-Zn SOD gene under the control of the lac promoter. HeLa, J774.A1, and HEK293T cell lines were purchased from the American Type Culture Collection and cultured according to the method described previously [20].

### Bone marrow derived macrophages (BMDMs) culture

The bone marrow cells of BALB/c mice were flushed from femurs and tibias with a 25-gauge needle. Red blood cells (RBCs) were lysed with RBC lysis buffer (0.16 M NH4Cl, 0.17 M tris (pH 7.65)) for 3 min at 37°C, and washed with PBS. Cells were cultured in complete α-MEM supplemented with 10% fetal bovine serum, 1% glutamine Pen-Strep, and M-CSF (10 to 20 ng/ml; Peprotech). After 2 days in culture with M-CSF, non-adherent bone marrow derived macrophages (BMDMs) were transferred to new plates at a density of 5 × 10^6^ cells per 10-cm dish, 2.5 × 10^5^ cells per well in a 24-well plate, or 1 × 10^5^ cells per well for a 96-well plate. BMDMs were cultured for an additional 2 to 5 days with M-CSF [21].

### Cell infection and survival assay

J774.A1, BMDMs or HeLa cells were plated in 24-well plates in complete tissue culture media without antibiotics at a concentration of 2.0 × 10^5^ cells per well and incubated overnight at 37°C with 5% CO_2_. The cells were infected with *B. abortus* strain 2308 in triplicate wells of a 24-well plate at a multiplicity of infection (MOI) of 100:1 by centrifuging bacteria onto cells at 400 g for 10 min at 4°C. Following 15 min of incubation at 37°C in an atmosphere containing 5% CO2, the cells were washed three times with αMEM to remove extracellular bacteria and incubated for additional 60 min in medium supplemented with 50 μg/ml gentamicin to kill extracellular bacteria. To monitor *Brucella* intracellular survival, infected cells were lysed with 0.1% Triton X-100 in phosphate-buffered saline (PBS) at certain time points and serial dilutions of lysates were rapidly plated onto tryptic soy agar plates to enumerate CFUs [22].

### Infection of BALB/c mice

*B. abortus* strain 2308 strains were grown on TSB and infection doses were prepared as described previously [11]. Briefly, 6-8weeks old BALB/c mice were infected via the intraperitoneal route with approximately 1 × 10^5^ *Brucella*. At 0, 5, 10 and 20 days post infection, 3 mice per experimental group were sacrificed according to the animal protocol of Jilin University. The spleens were harvested aseptically and homogenized in sterile PBS. Spleen homogenates serially were diluted 10-fold in sterile PBS and were rapidly plated onto tryptic soy agar plates to enumerate CFUs at each time point. Mean CFUs from each test group were determined, and the data were expressed as log_10_^CFU/mg spleen^.

### Recombinant DNA techniques

Myc-Sar1 or Flag- SOD constructs were made by sub-cloning the mouse Sar1 or *Brucella* SOD gene into the expression vector pcDNA3-Myc or pcDNA3-Flag by PCR, respectively. pBBR1MCS-2- SOD (K60R, D61A, and K63R), Flag-SOD (K60R, D61A, and K63R), Myc-Sar1 (T39G) or Myc-Sar1 (H79G) were generated with the QuikChange Site-Directed Mutagenesis kit (Stratagene), respectively. Plasmid Flag-Cu-Zn SOD was constructed by sub-cloning the Cu-Zn SOD gene into expression vector pcDNA3-Flag. In a similar way, His- Sar1 (T39G), Sar1 (H79G) or Cu-Zn SOD constructs were made in pQE-80L expression vector. The generated plasmids were confirmed by DNA sequencing [20].

To produce Cu-Zn SOD or CyaA with C-terminal 3Flag fusion proteins, Cu-Zn SOD or CyaA sequence was amplified using primers A (ggtaccatgatgaaggaaacagctatgaagtccttatttatt) and B (tctagattattcgatcacgccgcagg) with *B. abortus* genomic DNA as template, and primers C (tggtaccatgatgaaggaaacagctcagcaatcgcatcaggctggt) and primers D (gtctagactggcgttccacatgcgcccagcga) with *B. pertussis* genomic DNA as template, respectively. And then the PCR products were separately inserted in pV1900-rab2 digested by Asp718I and XbaI. The resulting SOD-3Flag and CyaA-3Flag fusions were extracted from the vector digested with Asp718I and BamHI and cloned in pBBR1MCS with the same restriction enzymes producing pBBR1MCS-Cu-Zn SOD-3Flag and pBBR1MCS-CyaA-3Flag, respectively. The generated plasmids were confirmed by DNA sequencing. PV1900-rab2 and pBBR1MCS were generously provided by Professor Xavier De Bolle (University of Namur, Belgium) [7]. The plasmid pBBR1MCS- SOD-3Flag-CyaA was constructed by PCR amplification of the catalytic portion of the CyaA gene (first 399 codons) from *B. pertussis* genomic DNA with primers E (cggtatcgatggatcccagcaatcgcatcaggctggt) and F (attcgatatcggatccctggcgttccactgcgcccagcga), and cloning into the BamHI-digested pBBR1MCS-Cu-Zn SOD-3Flag using in-fusion, and was confirmed by sequencing [10].

### Recombinant protein expression and purification

*E. coli* strain (JM109) was used as the host for expression of recombinant proteins. Bacteria were grown at 37°C in LB medium (20 mg/ml ampicillin) to an OD600 of 0.5, induced with 1.0 mM IPTG, and cultivated for an additional 3 h at 37°C. Cells were harvested and sonicated 10 cycles of 30 s and spun at 12,000 *g* for 30 min. Supernatant was filtered with a 0.45-mm filter. N-terminal 6× His-tagged Cu-Zn SOD, Sar1(H79G)and Sar1(T39N) expressed with the pQE-80L vector were purified by using Ni-NTA agarose beads (QIAGEN) followed by Hitrap Q HP ion exchange chromatography (GE Healthcare). Recombinant proteins were kept in a buffer containing 25 mM HEPES (pH 7.4) and 150 mM NaCl. The protein content was assayed by BCA protein assay reagent (Pierce, USA) and protein purity was examined by Coomassie blue staining of SDS-PAGE gels [23].

### Western blotting

Cells were lysed in ice-cold radioimmunoprecipitation assay (RIPA) buffer. The protein content was assayed by BCA protein assay reagent (Pierce, USA). Twenty micrograms of protein was loaded to SDS-PAGE and then transferred to PVDF membrane. Membrane was incubated with a 1:1000 dilution of primary antibody, followed by a 1:2000 dilution of horseradish peroxidase-conjugated secondary antibody. Protein bands were visualized by ECL (GE Healthcare). The band densities were measured by densitometry (model GS-700, Imaging Densitometer; Bio-Rad). The background was subtracted from the calculated area [20].

### Immunoprecipitation

Cells were lysed in ice-cold RIPA buffer for 20 min followed by centrifugation at 16,000*g* for 10 min at 4 °C to remove insoluble materials. The resulting supernatants were subjected to immunoprecipitation reactions with the indicated antibodies and protein A- or protein G-agarose (Sigma). The beads were extensively washed with RIPA buffer, and the proteins were separated by SDS-PAGE. The proteins were transferred to PVDF membrane by Western blotting, incubated with the appropriate antibodies, and visualized by ECL (GE Healthcare).

### Immunofluorescence staining

Cells were fixed with 1–2 % paraformaldehyde and permeabilized with 0.05% saponin in PBS for 30 min at room temperature. After being blocked with antibody (2.4G2) for 1 h at room temperature, the slides were incubated with primary antibody overnight at 4°C. Cells were washed with PBS and incubated for 1 h at room temperature with secondary antibody that is conjugated with a fluorescent dye. Finally, the cells were washed with PBS three times (for 10 min each), and the immunofluorescence staining was visualized under Zeiss LSM510 confocal microscope. The images were analyzed with LSM confocal software. The primary antibodies used for immunofluorescence microscopy were c-Myc (Cell Signaling) rabbit mAb or mouse anti-Flag^*®*^ M2 antibody (Sigma). The secondary antibodies were goat anti-rabbit IgG (H + L) secondary antibody, Alexa Fluor^®^ 488 conjugate (Invitrogen), and donkey anti-rabbit IgG secondary antibody, Alexa Fluor^®^ 594 conjugate (Invitrogen).

### CyaA translocation assay

Translocation of Cu-Zn SOD–CyaA into host cells was assayed using the CyaA fusion approach. After infection of J774.A1 cells (moi 100:1) for the indicated times in 96-well plates (10^5^ cells per well), cells were gently washed five times with PBS and lysed. Intracellular cAMP levels were determined by cAMP enzyme immunoassay kit from Sigma-Aldrich Corporation LLC as described by the manufacturer [9].

### Electron microscopy

*B. abortus* strain 2308 or the strain overexpressed Cu-Zn SOD was cultured in TSB to an OD600 of 1.0 (or to the late log phase). The rest procedures were performed as previous study described [4]. Briefly, bacteria were fixed in an ice-cold solution of 4% formaldehyde and 2.5% glutaraldehyde. And then the samples were then fixed in ice-cold 1% OsO4 phosphate buffer. Finally, the samples were embedded in Spur medium and stained with uranium acetate and lead citrate and examined in a transmission electron microscope.

### Detection of overexpressed Cu-Zn SOD release in bacteriological culture medium

*B. abortus* strain 2308 or the strain overexpressed Cu-Zn SOD was cultured in TSB to an OD600 of 1.0. Bacterial pellet and supernatant were separated by centrifugation (4000 g, 15 min). The supernatants were mixed with cold trichloroacetic acid (TCA), a final concentration of 20%, incubated at 4°C overnight. The pellet was separated by centrifugation (15,000 *g*, 30 min). Concentrated supernatant pellets or bacterial pellets were re-suspended in SDS-PAGE loading buffer. The protein constituents were resolved by SDS-PAGE. The gels were transferred to PVDF membranes for Western blot analyses or stained with Coomassie brilliant blue [9].

### Phospholipase D (PLD) assay

PLD (sigma) activity was determined as previously described [17]. Briefly, Microsome membranes were incubated in the presence of GTP, His-Sar1(T39G), His-Sar1(H79G) or His- Cu-Zn SOD for 60 min at 37°C, in the presence of radiolabeled substrate lipsome composed PE and PC (sigma) ( molar ratio 4:1).The reaction of assay was terminated at 60 min by the addition of chloroform: methanol. Lipids were extracted and dried, and PET formation was determined on TLC plates.

### Statistical analysis

Statistical analysis was performed using SPSS10.0 software. Data are expressed as mean ± SEM. The statistical significance of differences was evaluated by using one-way ANOVA followed by the Student’s *t* test. *P* < 0.05 was considered statistically significant.

## SUPPLEMENTARY MATERIALS FIGURES


