# Supplementary material for: Overexpression of Cu-Zn SOD in Brucella abortus suppresses bacterial intracellular replication via down-regulation of Sar1 activity
Source: Oncotarget. 2018 Jan 10;9(11):9596–607. doi: 10.18632/oncotarget.24073 (PMC5839387; doi:10.18632/oncotarget.24073)
Supplement: Supplementary file 1 [file oncotarget-09-9596-s001.pdf]

Overexpression of Cu-Zn SOD in *Brucella abortus* suppresses bacterial intracellular replication via down-regulation of Sar1 activity

SUPPLEMENTARY MATERIALS

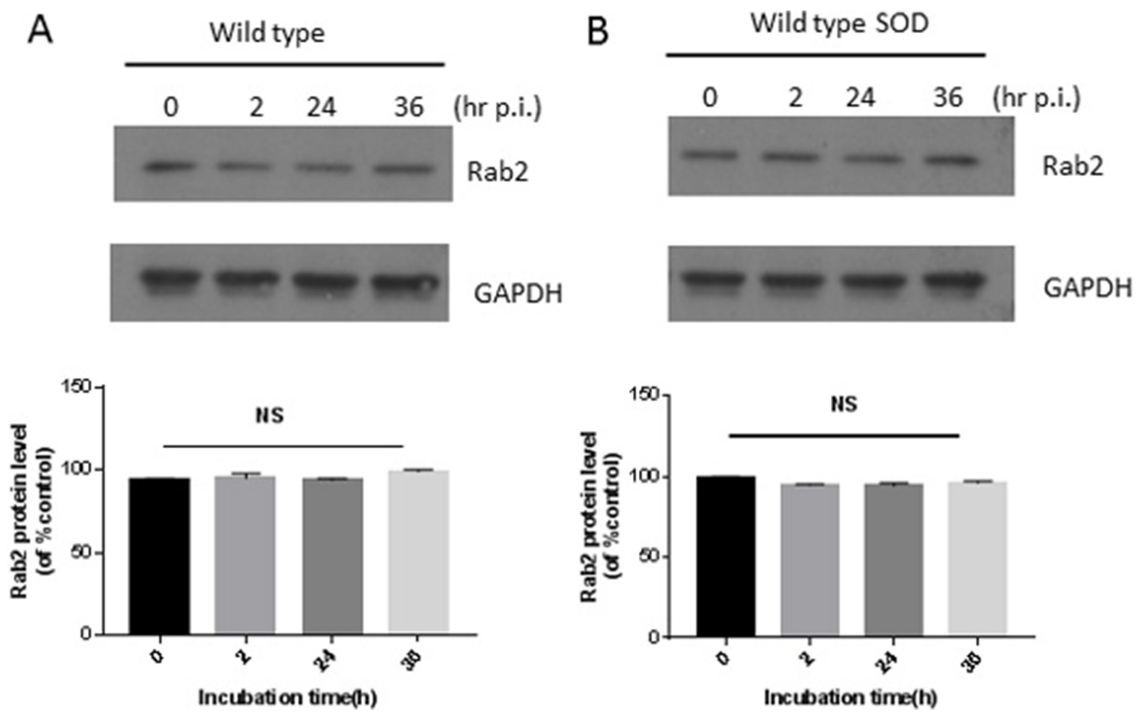

Supplementary Figure 1: Infection with B. abortus or wild type SOD do not mediate Rab2 expression. BMDM cells were infected with *B. abortus* (**A**) or wild type SOD (**B**) as indicated times. Cell lysates were collected and analyzed by Western blotting to determine the protein levels of Rab2. GAPDH was used for normalization. The blot is a representative of 3 independent experiments. The quantitative data for Rab2 expression are shown under Western blotting data. Data are expressed as mean ± SEM ($n = 3$).$^{*}P < 0.05$ VS control. NS indicates not significant.

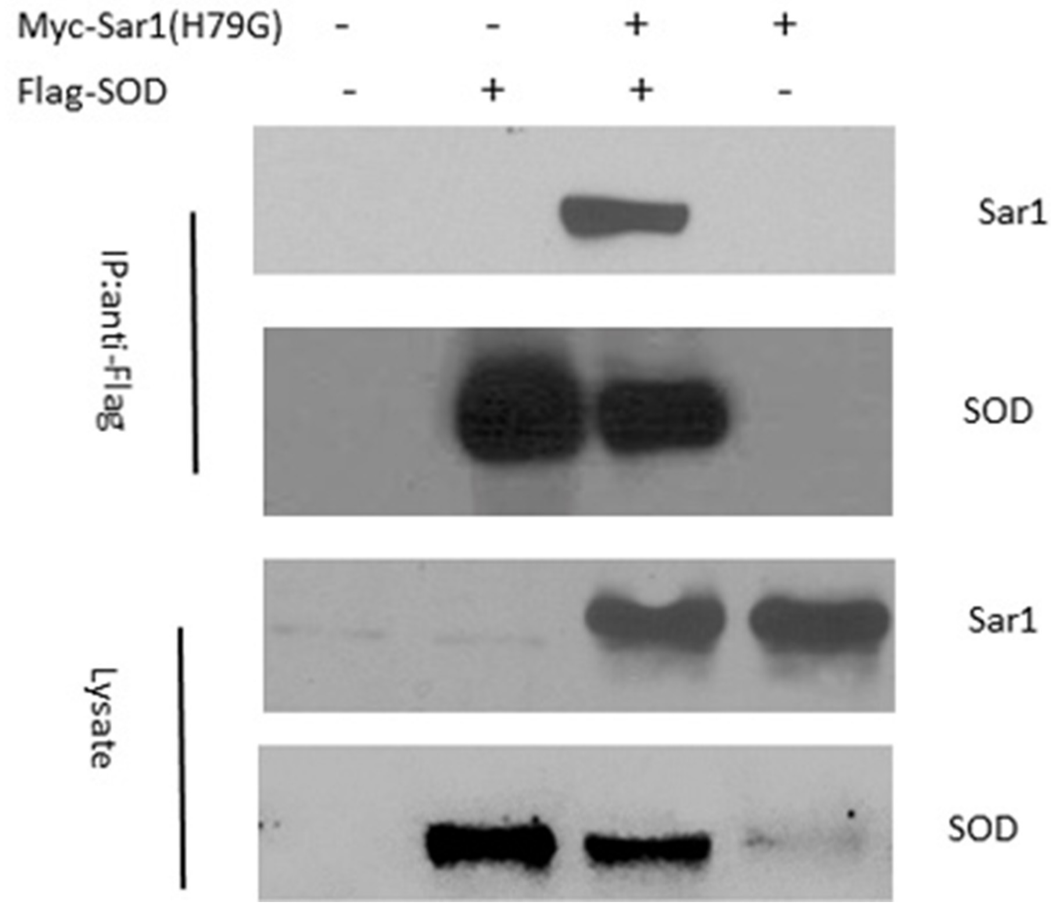

Supplementary Figure 2: B. abortus Cu-Zn SOD can bind to Sar1(H79G). Flag-tagged *B. abortus* Cu-Zn SOD, Myc-Sar1(H79G), or empty vector was expressed in HEK293 cells. Cu-Zn SOD was immunoprecipitated with anti-Flag antibody. Co-precipitated Cu-Zn SOD or Sar1(H79G) were detected by immunoblotting with anti-Flag or anti-Myc antibody (upper panel). The expression level of SOD or Sar1 were detected by immunoblotting the whole cell lysates with anti-Flag and anti-Myc antibodies (down panel), respectively.

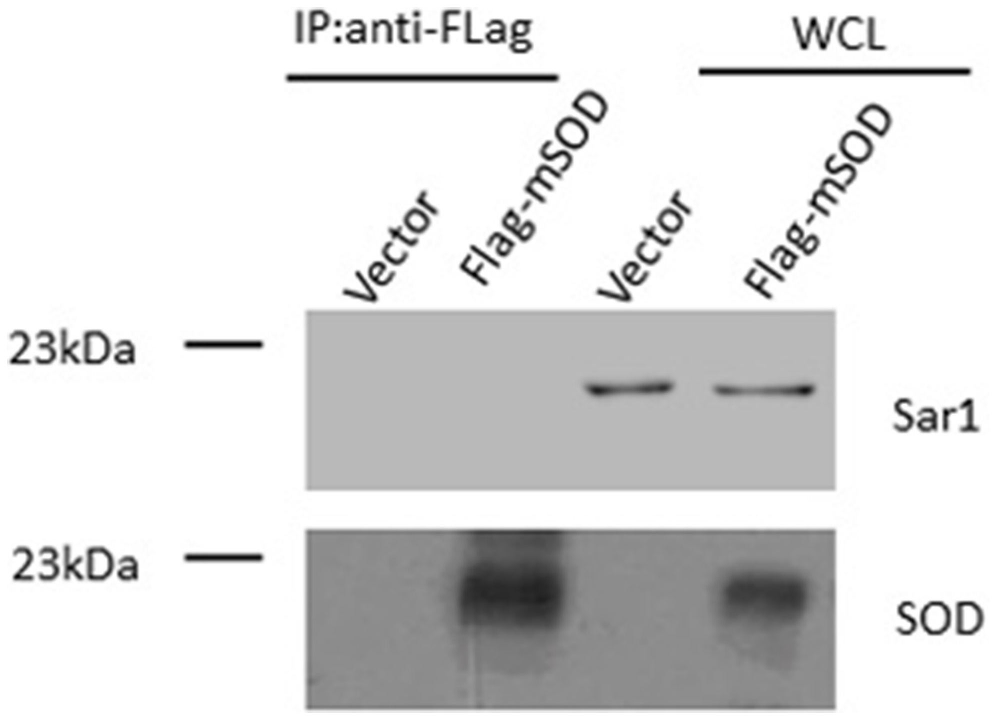

Supplementary Figure 3: The mutant version of Cu-Zn SOD (K60R, D61A, and K63R) cannot bind Sar1. Flag-tagged *B. abortus* Cu-Zn SOD(K60R, D61A, and K63R), or empty vector was expressed in HEK293 cells. Cu-Zn SOD was immunoprecipitated with anti-Flag antibody. Co-precipitated Cu-Zn SOD or Sar1 was detected by immunoblotting with anti-Flag or anti-Sar1 antibodies, respectively. WCL indicates whole cell lysates; mSOD means Cu-Zn SOD(K60R, D61A, and K63R).

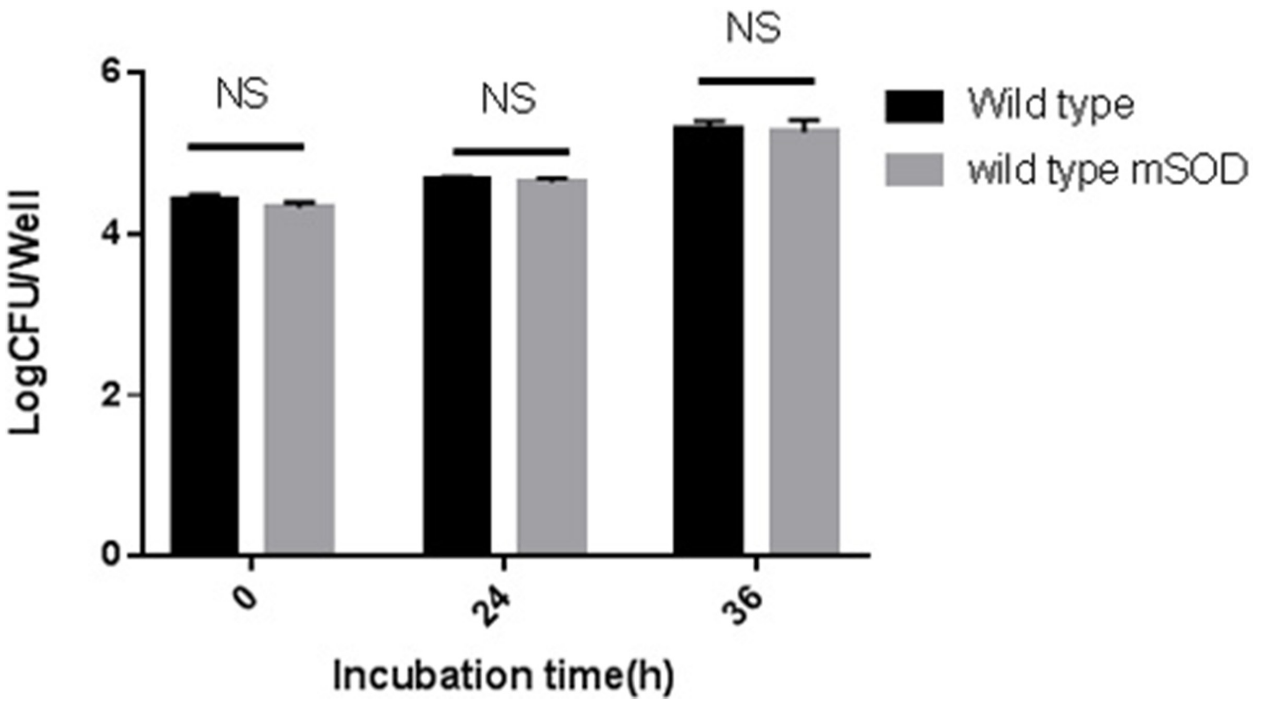

Supplementary Figure 4: Overexpressed Cu-Zn SOD (K60R, D61A, and K63R) in B. abortus does not affect bacterial intracellular growth. (**A**) BMDM cells were infected with *B. abortus* (wild type), or expressing Cu-Zn SOD (K60R, D61A, and K63R) strains as indicated times. The number of viable bacteria was determined by counting CFU. (**B**) BALB/c mice were injected intraperitoneally with 10^5 wild type, or Cu-Zn SOD (K60R, D61A, and K63R) strains. The number of viable bacteria was determined by counting CFU as the mean of LOG_{10}^{CFU} per milligram of spleen. mSOD means Cu-Zn SOD(K60R, D61A, and K63R).
